# X-ray Structure Analysis of a Novel 1C Metabolism Pathway in *Sphingobium lignivorans* SYK-6: Cooperative Function of LigM and S6MTHFR

**DOI:** 10.1063/4.0000986

**Published:** 2025-10-27

**Authors:** Toshiya Senda, HongYang Yu, Naofumi Kamimura, Eiji Masai

**Affiliations:** 1 KEK, Tsukuba, Ibaraki, Japan; 2 Nagaoka University of Technology, Nagaoka, Niigata, Japan

## Abstract

*Sphingobium lignivorans* SYK-6 (formerly *Sphingobium* sp. strain SYK-6) is a model bacterium widely studied for its ability to catabolize lignin- derived aromatic compounds (Masai et al., 2007). As lignin is one of the most abundant yet underutilized components of plant biomass, understanding the enzymatic systems employed by such microbes holds great potential for sustainable bioprocessing. SYK-6 can grow on lignin- derived low-molecular-weight aromatics (low-molecular-weight lignins; LMW lignins), such as vanillate, making it a prime system for investigating how microbes break down and utilize lignin.

In addition to its ability to catabolize lignin-derived aromatics, SYK-6 features a distinctive central metabolism: it cannot grow on glucose and lacks the glycine cleavage system, which in many organisms supplies 1C units in the form of 5,10- methylenetetrahydrofolate (5,10-CH_2_THF). Instead, SYK-6 utilizes a non-canonical 1C pathway involving two key enzymes. LigM, a tetrahydrofolate (THF)-dependent *O*-demethylase likely evolved from the T-protein of the glycine cleavage system, transfers the methyl moiety of the methoxy group from vanillate to THF, forming 5- methyltetrahydrofolate (5-CH_3_THF). To convert this into another form of 1C donor, SYK-6 employs S6MTHFR, a methylenetetrahydrofolate reductase homolog that catalyzes the reverse of the typical reaction—oxidizing 5-CH_3_THF to 5,10-CH_2_THF. This reaction effectively compensates for the absence of the glycine cleavage system. Structural and mutational analyses already revealed that LigM has evolved specific adaptations for recognizing small aromatic substrates, reflecting its specialized role in lignin-derived 1C metabolism (Harada *et al.*, 2017). Recently, we have performed biochemical and structural analysis of S6MTHFR (Yu *et al.*, 2025). X-ray crystallography of S6MTHFR revealed that it retains the TIM-barrel fold typical of MTHFRs but exhibits unique structural features near the cofactor-binding site that enable this reversed catalytic activity. Biochemical assays confirmed that S6MTHFR oxidizes 5- CH_3_THF, thereby supplying 5,10-CH_2_THF as a biosynthetically active 1C donor. This enzymatic adaptation allows SYK-6 to maintain 1C metabolism without access to conventional glycine-cleavage pathways. LigM and S6MTHFR form a tightly coupled pathway that functionally replaces the glycine cleavage system. This enables SYK-6 to sustain essential folate- mediated processes without relying on the glycine cleavage system. The two enzymes represent a novel evolutionary solution to life in nutrient-restricted, LMW-lignin-rich environments.

These findings underscore how structural adaptation and enzymatic innovation can give rise to entirely new metabolic frameworks. The LigM–S6MTHFR module exemplifies an alternative 1C unit acquisition strategy optimized for lignin degradation and opens up avenues for engineering microbial hosts to convert lignin-derived carbon into industrially relevant products. Furthermore, large-scale genome database analysis has revealed that many other bacteria possess similar gene sets, suggesting that this type of metabolic strategy may be widespread in lignin-degrading microbial communities (Yu *et al.*, 2025).
